# To Bend or Not to Bend: Revealing the Stereoelectronic Origin of the Distorted *sp* Carbon in Isocyanates

**DOI:** 10.1021/acs.jpca.5c02484

**Published:** 2025-08-13

**Authors:** Lucas Araujo, Felipe Fantuzzi, Thiago M. Cardozo, Lars V. Schäfer

**Affiliations:** † Center for Theoretical Chemistry, 9142 Ruhr University Bochum, Universitätsstr. 150, 44801 Bochum, Germany; ‡ Chemistry and Forensic Science, School of Natural Sciences, 28125 University of Kent, Park Wood Rd, Canterbury CT2 7NH, U.K.; § Instituto de Química, 341411 Universidade Federal do Rio de Janeiro, Av. Athos da Silveira Ramos, 149, CT, A-622, Cid. Univ., Rio de Janeiro, RJ 21941-909, Brazil

## Abstract

Isocyanates, conventionally depicted as R–N=C=O, exhibit a puzzling deviation from the expected linear geometry of *sp*-hybridized carbon centers—a structural bending that significantly influences their reactivity. In this work, we present a comprehensive theoretical investigation into the structural, electronic, and vibrational properties of isocyanates using density functional theory (DFT) and wave function-based methods. The chemical structure of isocyanates is explored through intrinsic bond orbitals (IBOs), spin-coupled generalized valence bond (SCGVB) theory, and interference energy analysis (IEA) based on SCGVB calculations. Within the valence bond framework, we show that the N=C and C=O bonds in HNCO are best described as bent (or “banana”) bonds, as the conventional representation using orthogonal σ and π components leads to physically unreasonable results. The IEA further reveals that the two bent bonds in C=O are not equivalent. This difference originates from the asymmetric electronic environment induced by the neighboring nitrogen lone pair, which weakens one of the bonds in C=O and induces observed NCO bending. By reasoning in terms of the two dominant resonance structures, we show that different substituents can favor one form or the other, depending on their nature. These results provide a clear rationale for the distinctive electrophilic behavior of isocyanates and also contribute to a deeper understanding of the so-called “bent *sp* carbon.”

## Graphical abstract

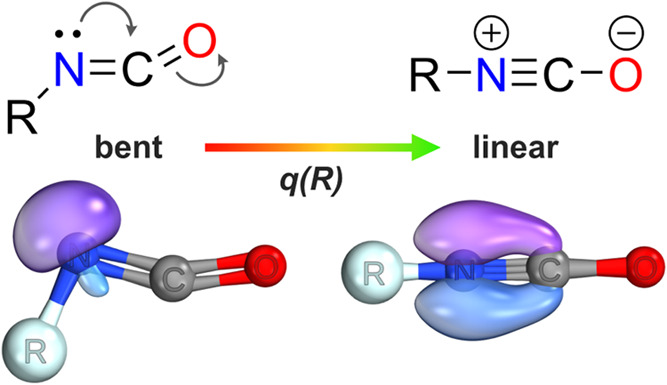

## Introduction

Molecules containing the isocyanate (NCO) group are generally termed isocyanates (RNCO). The simplest member, HNCO, is a known atmospheric pollutant primarily formed during biomass and fossil fuel combustion.^1^ Additionally, HNCO is of significant interest in astrochemistry and astrobiology because of its composition—it contains four of the six key elements (CHNOPS) essential for life^2,3^ —and because of its presence in diverse astronomical environments, including molecular clouds,^4^ hot molecular cores,^5^ solar-type protostars,^6,7^ and comets.^8^ As a tetraatomic molecule with 16 valence electrons, HNCO is isoelectronic with other chemically relevant species such as HN_3_ and HOCO^+^.^9–11^ Moreover, HNCO and its structural isomers—fulminic acid (HCNO), isofulminic acid (HONC), and cyanic acid (HOCN)—have been investigated since the 16th century, with isocyanic acid being the most stable among them.^12,13^ Since the discovery of polyurethanes by Bayer and co-workers in 1937,^14^ isocyanates have become major industrial chemicals due to their high reactivity with alcohols.^15^ In medicinal chemistry, the inherent reactivity of the NCO group is exploited to generate stable urea and carbamate derivatives, which serve as key structural motifs in a variety of bioactive molecules with applications ranging from bioimaging^16^ to anticancer and antimicrobial therapies.^17,18^ Despite their importance, the fundamental electronic and structural properties of the isocyanate group remain a subject of debate, particularly the deviation of the *sp*-hybridized carbon from the expected linear geometry.

Traditional chemical representations depict *sp*-hybridized carbons as strictly linear, as seen in, for example, CO_2_, acetylene (HCCH), and hydrogen cyanide (HCN), where the π-bonding framework aligns with the linear *sp*-hybridization model.^19–21^ However, there are exceptions. For instance, allenes, despite having a linear C=C=C core, can exhibit bent geometries.^22^ Similarly, carbyne chains and molecules with triply bonded third-row elements also exhibit *trans*-bent geometries due to the dominance of the σ-framework over the weaker π-bonds.^23–25^ In R–N=C=O, the expected linear *sp*-hybridized carbon deviates from this norm, exhibiting a bent geometry between nitrogen and oxygen.

Historically, this deviation was attributed to resonance structures. Over 80 years ago, Eyster and Gillette^26^ proposed that this bending arises from a combination of three ionic resonance structures: R–N=C=O, R–N^+^≡C–O^–^, and R–N^–^–C≡O^+^. Caraculacu and Coseri later suggested alternative resonance structures, namely, R–N=C=O, R–N=C^+^–O^–^, and R–N^–^–C^+^=O, emphasizing electron density distribution and reactivity.^27^ Later, Delebecq et al.^15^ highlighted the lack of consensus regarding electron density and partial charges within the isocyanate group. More recently, Faller and Nguyen discussed the wide range of RNC angles in isocyanates using terms of two resonance structures, R–N=C=O and R–N^+^≡C–O^–^, based on empirical observations.^28^ Despite these extensive resonance-based interpretations, a clear and universally accepted explanation for the characteristic nonlinear (or quasi-linear) NCO angle in isocyanates remains, to the best of our knowledge, elusive in the literature.

While high-level *ab initio* computations and microwave spectroscopy provided detailed information on the structure and electronic properties of HNCO,^13,29–31^ uncertainties remain regarding charge distribution, particularly for nitrogen and oxygen. Qualitative interpretations suggest similar partial charges on nitrogen and oxygen and a reduced electron density at carbon, consistent with observations for NCO^–^.^32^ Understanding these subtleties is essential because the bent geometry of isocyanates directly influences their electrophilic reactivity. These compounds undergo nucleophilic addition at the N=C bond, forming ureas, carbamates, and other derivatives, while the C=O bond is significantly less reactive.^33,34^ The efficiency of such reactions is critically governed by the electron distribution across the N=C=O moiety, emphasizing the need for a comprehensive understanding of the underlying stereoelectronic factors.

In this work, we investigate the origin of the quasi-linearity of isocyanates using quantum chemical calculations based on density functional theory (DFT) and wave function-based methods. In particular, we examine the electronic structure of HNCO and related systems in various structural configurations by employing localized orbital sets derived from both molecular orbital (MO) and valence bond (VB) frameworks—specifically, the intrinsic bond orbital (IBO)^35,36^ and spin-coupled generalized valence bond (SCGVB)^37^ methods, respectively. The SCGVB approach self-consistently yields a bent-bond description, whereas the IBO method naturally converges to the σ–π separation picture. The longstanding ambiguity in representing multiple bonds—first noted by Slater^38^ and Pauling^39^—is addressed here by treating these two approaches as complementary. We contextualize the SCGVB results using the interference energy analysis (IEA),^40,41^ which employs the generalized product function energy partitioning (GPF-EP)^42^ approach to quantify the quantum interference contributions of individual electron pairs. Complementary vibrational analyses, with an emphasis on the bending mode, further reveal the interplay between orbital interactions and structural preferences. Taken together, this comprehensive investigation unravels the stereoelectronic origins of isocyanate quasi-linearity and clarifies how orbital interactions dictate structural distortions and govern chemical reactivity.

## Computational Details

All geometry optimizations, vibrational frequency calculations, and IBO analyses, including intrinsic atomic orbital partial charges (hereafter referred to as IBO charges), were performed using ORCA 6.0.1.^43^ Most molecular structures were obtained using the ωB97M-D4 ^44^ density functional with the def2-TZVPP basis set,^45^ which offers a balanced treatment of valence and polarization effects well-suited to DFT. Analytical gradients and Hessians were employed in all DFT calculations. For reference geometries, CCSD­(T) coupled-cluster calculations were carried out^46^ with the aug-cc-pVTZ basis set,^47^ which includes diffuse functions essential for accurate correlation treatment, and the frozen-core approximation. Numerical gradients and Hessians were used for the CCSD­(T) calculations. All stationary points were confirmed as minima by the absence of imaginary vibrational frequencies. The CCSD­(T)-optimized geometries and corresponding vibrational frequencies are provided in Tables S1 and S2 of the Supporting Information (SI).

All SCGVB calculations were performed using VB2000 ^48^ software as implemented in GAMESS (version September 30, 2022 R2).^49^ We considered both the perfect-pairing (PP) and strong-orthogonality (SO) approximations, which are referred to simply as SCGVB-PP. These were achieved by expressing the SCGVB wave function as a generalized product function (GPF).^50^ Briefly, a GPF is constructed by partitioning electrons into distinct groups and describing the overall system as a product of antisymmetrized, strongly orthogonal wave functions for each group

$$\Psi_{\mathrm{GPF}}\left({\mathrm{x⃗}}_{1},\cdot \cdot \cdot ,{\mathrm{x⃗}}_{N}\right)=A\left[\begin{array}{l} \Psi^{\left(1\right)}\left({\mathrm{x⃗}}_{1},\cdot \cdot \cdot ,{\mathrm{x⃗}}_{n_{1}}\right)\\ \Psi^{\left(2\right)}\left({\mathrm{x⃗}}_{n_{1}+1},\cdot \cdot \cdot ,{\mathrm{x⃗}}_{n_{1}+n_{2}}\right)\\ \cdots \\ \Psi^{\left(N\right)}\left({\mathrm{x⃗}}_{n_{N-1}+1},\cdot \cdot \cdot ,{\mathrm{x⃗}}_{N}\right) \end{array}\right]$$ 1

where *x⃗*_*i*_ represents the spatial and spin coordinates of the *i*-th electron and Ψ^(*k*)^ are antisymmetrized wave functions for groups of *n*_*k*_ electrons. The antisymmetrizing operator A ensures proper exchange symmetry among groups.

The reliability of SCGVB-PP was evaluated via SCGVB(8).SCGVB-PP calculations (see SI for details), which include all spin functions for eight electrons and remove strong orthogonality within the SCGVB(8) group. These were compared with CASSCF­(8,8)/cc-pVTZ calculations; representative active orbitals for the latter are shown in Figure S1. The results confirm that PP spin-coupling remains dominant upon expansion. Energy differences between distinct HNCO structures (vide infra) obtained with SCGVB-PP are in close agreement with those from both CASSCF­(8,8) and SCGVB(8).SCGVB-PP (Table S3). Gallup-Norbeck coefficients^51^ (Table S4) further support the validity of SCGVB-PP, showing exclusive contribution from the PP spin function in all cases.

In the GPF-EP method,^42^ the GPF first- and second-order reduced density matrices are partitioned into quasi-classical and interference contributions.^52–56^ For example, the one-electron density (ρ) is expressed as the sum of the quasi-classical (ρ^QC^) and interference (ρ^I^) densities. For a single electron group *m*, the quasi-classical density is expressed as

2 $$\rho_{m}^{\mathrm{QC}}\left(\mathrm{r⃗}\right)=\sum_{j=1}^{N_{m}}{\left[\phi_{j}^{\left(m\right)}\left(\mathrm{r⃗}\right)\right]}^{2}$$

with *N*_*m*_ being the number of electrons in group *m* and ϕ_*j*_^(*m*)^(*r⃗*) being the corresponding group orbitals. In contrast, the interference density is defined as

3 $$\rho_{m}^{I}\left(\mathrm{r⃗}\right)=\sum_{j\neq k}^{N_{m}}{\left\langle j,k\right\rangle }_{m}p\left(j|k\right)$$

where ⟨*j, k*⟩_*m*_ denotes the interference term associated with orbitals ϕ_*j*_^(*m*)^ and ϕ_*k*_^(*m*)^ and *p*(*j*|*k*) represents the first-order reduced density matrix expressed in the orbital basis. Specifically, ⟨*j, k*⟩_*m*_ is given by

4 $${\left\langle j,k\right\rangle }_{m}=\phi_{j}^{\left(m\right)}\left(\mathrm{r⃗}\right)\phi_{k}^{\left(m\right)}\left(\mathrm{r⃗}\right)-\frac{1}{2}\xi \left(j,k\right)\left\{{\left[\phi_{j}^{\left(m\right)}\left(\mathrm{r⃗}\right)\right]}^{2}+{\left[\phi_{k}^{\left(m\right)}\left(\mathrm{r⃗}\right)\right]}^{2}\right\}$$

with ξ­(*j, k*) being the overlap integral between the two orbitals.

The total energy of the system, *E*[TOT], is partitioned into several components

5 $$E\left[\mathrm{TOT}\right]=\underset{E\left[\mathrm{QC}\right]}{\underset{︸}{E\left[\mathrm{REF}\right]+E\left[X\right]}}+\underset{E\left[\mathrm{INT}\right]}{\underset{︸}{E\left[I\right]+E\left[\mathrm{II}\right]}}$$

where *E*[REF] is the reference energy, *E*[X] accounts for the intergroup exchange interaction due to the antisymmetry of the GPF, and *E*[I] and *E*[II] represent the first- and second-order interference energies, respectively. The complete formalism of the GPF-EP method can be found elsewhere.^42^ The symmetry-corrected quasi-classical energy, *E*[QC], and the total interference contribution, *E*[INT], are defined in eq 5. In the SCGVB calculations presented here, the valence electrons were distributed in GPF groups of two electrons, while the core electrons were treated as a single Hartree–Fock group. It should be noted, therefore, that throughout this work the SCGVB wave functions are, in fact, SCGVB-PP, and the two labels are used interchangeably.

## Results and Discussion

### Rationale for HNCO

The radical NCO^•^ and the anion NCO^–^ are both linear but exhibit different bonding patterns.^57,58^ While the radical is characterized by two double bonds (N=C=O^•^), the anion is typically depicted with one triple bond and one single bond (N≡C–O^–^). Although resonance structures can be drawn for both systems to aid in chemical reasoning, they are generally rationalized by a central *sp-*hybridized carbon that justifies the linear geometry. However, upon attaching a substituent, such as hydrogen, to the nitrogen of the radical or protonating the anion, the resulting HNCO adopts a bent geometry. This bending, particularly along the H–N–C angle, can be attributed to repulsive interactions between the nitrogen lone pair and the newly formed H–N bond. Notably, the structure is not only bent at the nitrogen but adopts an overall *trans*-bent configuration, where the NCO moiety becomes quasi-linear.

When comparing the valence bond Lewis (vbL) diagrams^59–61^ of the resonance structures for the two isoelectronic species, NCO^–^ and HNCO, we find that while the structure of NCO^–^ is well-described by Scheme 1(A), this is not the case for HNCO. In HNCO, in order for the NC moiety to support two equivalent π-bonds, a zwitterionic structure must be invoked, introducing charge separation in the neutral molecule, which is presumably a higher-energy configuration. Alternatively, if the nitrogen adopts an *sp* ^2^ hybridization (rather than being partially positively charged and *sp*-hybridized), one π electron pair forms a bond while the other becomes a lone pair, corresponding to the neutral structure shown in Scheme 1(B). However, the protonated *sp* ^2^ nitrogen results in two nonequivalent resonance structures, in contrast to the two symmetry-equivalent N=C π-bonds in NCO^–^. This distinction arises from the symmetry of the π-bonds: in NCO^•^ and NCO^–^, the two N=C π-bonds are equivalent in both the *xz* and the *yz* planes. In contrast, for HNCO, each configuration involves a lone pair and a π-bond in different planes; however, they are not equivalent due to the presence of the H–N bond in one of the planes—a difference that can also be rationalized using the VSEPR model.^62^ The two chemical structures shown in Scheme 1(A),(B) can be considered as limiting models for the chemical structures of isocyanates.

**Scheme 1:**
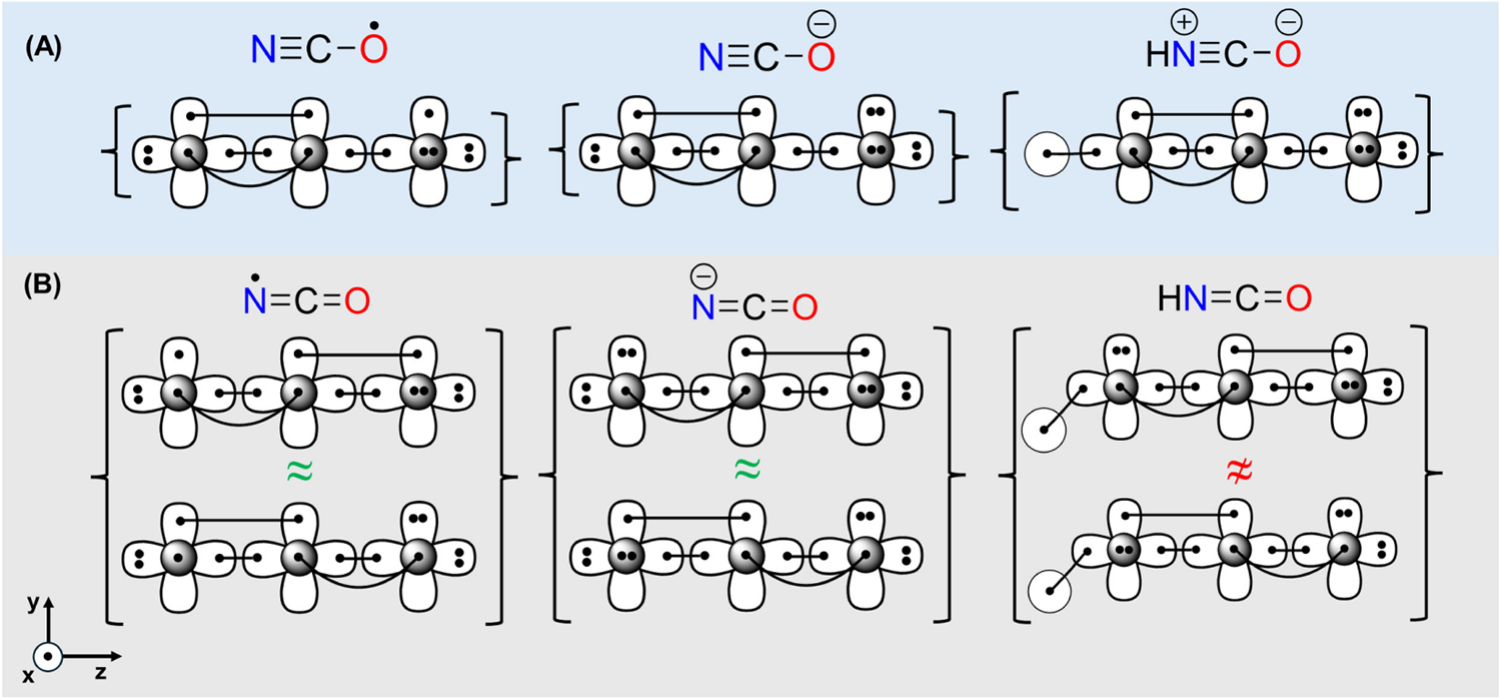
Comparison between the Resonance Structures and vbL Diagrams of NCO^·^, NCO^–^, and HNCO^a^

a (A) Resonance structure with a triple bond. (B) Resonance structure with two double bonds. The braces indicate the corresponding covalent resonance structures.

If the above hypothesis is correct, substituting the hydrogen atom with an electron-donating group should stabilize the resonance structure (A) of HNCO (Scheme 1), making the isocyanate resemble the free anion, with the lone pair converting into a π-like orbital. Conversely, replacing hydrogen with an electron-withdrawing group should stabilize the lone pair, favoring resonance structure (B). The asymmetry present in the resonance structures of (B) for HNCO, but absent in NCO^–^, may explain the bent geometry of the NCO moiety in HNCO and isocyanates, in general. In contrast, the preserved symmetry in NCO^–^ (B) contributes to its linearity.

To test this hypothesis, we examined the stereoelectronic differences in HNCO, focusing on three distinct structures: the bent, fully optimized HNCO featuring a kinked *sp* carbon (**HNCO** ^**bent**^); a bent H–NCO structure with a constrained linear NCO moiety (**HNCO** ^**bl**^); a fully constrained linear HNCO (**HNCO** ^**lin**^). Furthermore, since the observed kinks appear to depend on the nature of the substituent bonded to the isocyanate group, we also examined additional RNCO structures, systematically varying R to analyze their effect on the stereoelectronic properties of RNCO.

### IBO Picture of HNCO

First, we analyzed the energy variation of the HNCO with respect to the HNC angle. For that, we performed calculations at the CCSD­(T)/aug-cc-pVTZ level of theory. The HNCO equilibrium bond lengths at this level are 1.007, 1.222, and 1.170 Å for H–N, N=C, and C=O bonds, respectively, with bond angles of 122.5° for H–N=C and 172.1° for N=C=O. These values are consistent with those obtained by Mladenović^13^ using CCSD­(T)/cc-pCV5Z (1.003, 1.214, and 1.164 Å, with angles of 123.4 and 172.4°) as well as with experimental data derived from microwave spectroscopy (0.9946, 1.2140, and 1.1664 Å, with angles of 123.9 and 172.6°).^64^

Next, we performed a relaxed scan of the HNC angle at the CCSD­(T) level, monitoring both the structural parameters and the IBOs, which were obtained through single-point energy calculations at each point of the relaxed CCSD­(T) relaxed scan. The scan in Figure 1(A) reveals the bent minimum of HNCO, and constraining the HNC moiety to 180° leads to a fully linear structure. The barrier to linearity in this case is 5.76 kcal/mol, which is in fair agreement with the value of 5.195 kcal/mol previously reported using CCSD­(T)/cc-pV5Z, including core correlations.^29^ These results suggest that as the HNC angle increases from approximately 120 to 180°, the hybridization of the nitrogen atom shifts from *sp* ^2^ to *sp*, in accordance with Coulson’s theorem,^65^ and the NCO angle approaches 180°.

**Figure 1:**
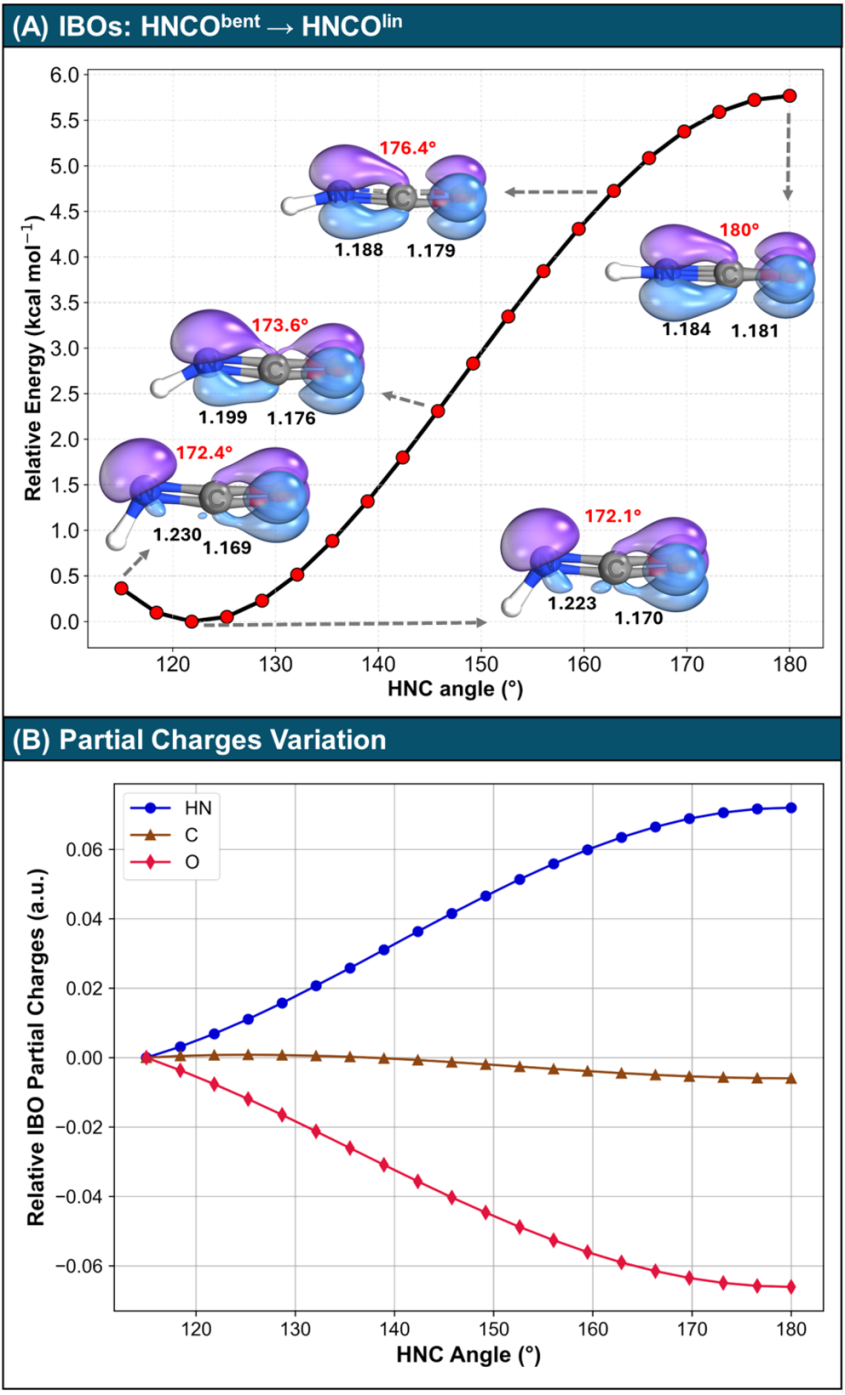
(A) Relaxed potential energy surface (PES) scan for HNCO calculated at the CCSD­(T)/aug-cc-pVTZ level. The HNC angle was varied from 115 to 180°. The NCO angle and the bond lengths (in Å) are given in red and black, respectively. Selected IBOs are visualized together with corresponding structural properties, namely, the N–C and C–O bond lengths and the NCO angle. Orbital iso-surfaces are plotted with a threshold of 50%. All orbital plots were made using IboView.^63^ (B) IBO partial charges (a.u.) along the PES scan, relative to the geometry with an HNC angle of 115°. The energies and charges plotted in this Figure are provided in Table S1.

Furthermore, the analysis of IBOs reveals a clear shift in the bonding scheme: the chemical structure transitions from H–N=C=O (Scheme 1(B)) to H–N^+^≡C–O^–^ (Scheme 1(A)). In this process, the C=O π-bond transforms into a polarized lone pair centered on the oxygen atom. This change in the bonding aligns with the observed bond length variations. Additionally, Figure 1(B) shows that the structural modifications correlate with changes in atomic partial charges. As the HNC and NCO angles approach 180°, the nitrogen lone pair is converted into a π-bond, increasing the positive character of the nitrogen. Concurrently, the C=O π-bond transforms into a lone pair, enhancing the negative character of the oxygen. This electronic reorganization is accompanied by a shortening of the N–C bond and a lengthening of the C–O bond, in line with the corresponding resonance structure.

### SCGVB Picture of HNCO

To investigate the influence of molecular geometry on the electronic structure, we performed constrained CCSD­(T) optimizations on HNCO. Starting from the fully optimized geometry (**HNCO** ^**bent**^), we first optimized it with the NCO group constrained to a 180° angle (**HNCO** ^**bl**^). Then, we enforced a fully linear geometry by also fixing the HNC group at 180° (**HNCO** ^**lin**^). The Cartesian coordinates of the optimized geometries are reported in Table S2. SCGVB calculations were performed on **HNCO** ^**lin**^ for both the rigid and relaxed structures, with the bond lengths preserved in the former case. No qualitative differences were observed between the relaxed and rigid structures regarding the interference energy analysis. Figure 2(A) presents the SCGVB orbitals for the minimum-energy bent geometry, the structure with the NCO angle constrained at 180°, and the linear structure. The results demonstrate a close agreement between SCGVB and IBOs concerning the electronic structure: both methods identify a bent minimum featuring N=C and C=O double bonds, while the linear structure exhibits an N≡C triple bond with three lone pairs localized on oxygen. These limiting cases align well with the resonance structures formulated in our working hypothesis (Scheme 1).

**Figure 2:**
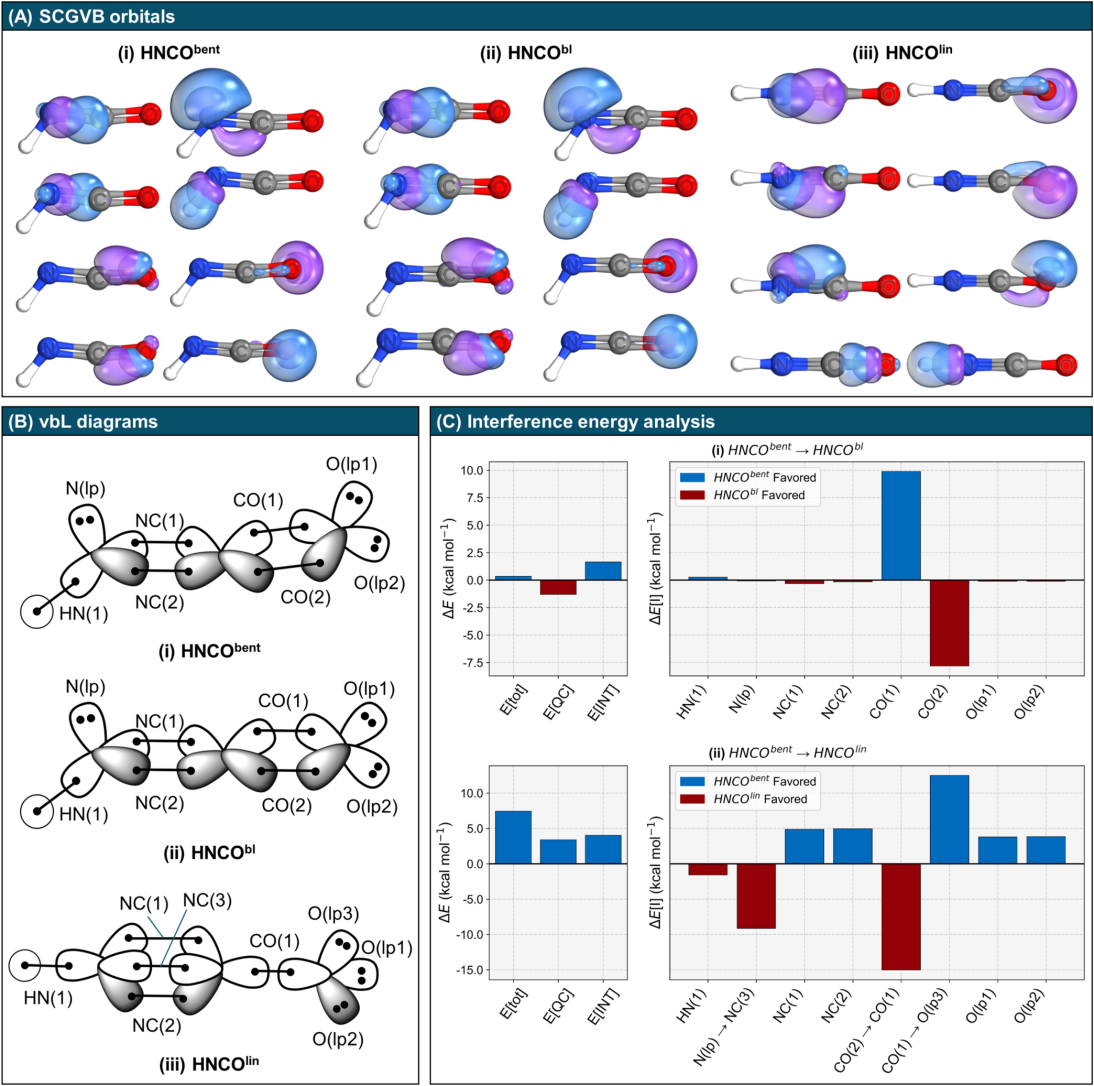
Valence bond picture of **HNCO** ^**bent**^, **HNCO** ^**bl**^, and **HNCO** ^**lin**^ along with GPF-EP analysis at the SCGVB-PP/cc-pVTZ//CCSD­(T)/aug-cc-pVTZ level. (A) SCGVB orbital pairs for (i) **HNCO** ^**bent**^, (ii) **HNCO** ^**bl**^, and (iii) orbitals of **HNCO** ^**lin**^, with an iso-surface threshold of 50% for visualization. (B) vbL diagrams for the bent and linear structures of HNCO, illustrating the orbital labeling conventions used in the analysis. (C) GPF-EP analysis of HNCO: (i) energy differences (left) and first-order interference energy differences (right) between **HNCO** ^**bent**^ and **HNCO** ^**bl**^; (ii) energy differences (left) and first-order interference energy differences (right) between **HNCO** ^**bent**^ and **HNCO** ^**lin**^.

Interestingly, for HNCO, the VB calculations inherently favor bent bonds (also known as banana bonds, equivalent bonds, τ-bonds, or ω-bonds). This was unexpected since it is well-known that SCGVB within the perfect pairing and strong-orthogonality approximations usually preserves the σ–π separation.^66–69^ The ambiguity in the representation of multiple bonds can be traced back to the work of Slater and Pauling. In the context of the Hartree–Fock model, these two representations are related by a unitary transformation and are therefore equivalent in energy. However, this equivalence does not hold in VB theory, where the wave function is not invariant to linear transformations of the active orbitals. In fact, most VB-based studies show that bent bonds are slightly lower in energy compared to σ–π bonds in most cases.^69–77^ These differences tend to become negligible as the number of active orbitals in the SCGVB function increases. However, attempts to apply a σ–π separated framework in HNCO led to significant energy discrepancies, suggesting that such an approach is unsuitable for this system. Further information can be found in the SI (Figure S2 and Table S3). Therefore, we proceed with the interference energy analysis of the bent bond.

From the SCGVB orbitals shown in Figure 2(A), we derived corresponding vbL diagrams for the three HNCO structures presented in Figure 2(B). These diagrams also include the orbital labeling conventions used in the interference energy analysis, the main results of which are highlighted in Figure 2(C). Specifically, the two upper plots display the energy partitioning for transformation (i) **HNCO** ^**bent**^ → **HNCO** ^**bl**^, while the bottom plots correspond to transformation (ii) **HNCO** ^**bent**^ → **HNCO** ^**lin**^. We begin by analyzing the left plots, which show energy partitioning into *E*[QC] and *E*[INT] contributions according to eq 5. For (i), the IEA results reveal that, in the absence of quantum interference, the molecule would preferentially adopt a linear NCO angle. This observation underscores the dominant role of quantum interference in inducing carbon *sp* bending, thereby enhancing the favorability of covalent interactions in the bent NCO structure.

The bottom left plot of Figure 2(C) shows that both *E*[QC] and *E*[INT] contribute to the stabilization of **HNCO** ^**bent**^ relative to that of **HNCO** ^**lin**^, with *E*[INT] being slightly larger. In both scenarios, (i) and (ii), the *E*[INT] contribution is primarily dominated by the first-order interference term, *E*[I], with second-order contributions *E*[II] having only a minor effect, as is typically observed (see Figure S3 and Table S5).^61,78,79^ Therefore, further interference analysis focuses on the first-order term *E*[I].

Next, we focus on the top and bottom right plots in Figure 2(C), which detail the partitioning of *E*[I] across various electron groups. In transformation (i), the analysis reveals that among all electron groups only the *E*[I] components corresponding to the bent bonds in C=O are affected by the linearization of the NCO motif. Moreover, the plot exposes a subtle asymmetry in these bonds, indicating that linearizing the NCO group does not render them equivalent, as might be inferred from a simple orbital inspection. Specifically, linearization causes CO(1) to be slightly more destabilized (favoring the **HNCO** ^**bent**^ configuration) than the stabilization observed in CO(2) (favoring the **HNCO** ^**bl**^ configuration), resulting in an overall destabilization of **HNCO** ^**bl**^ due to interference effects. This behavior arises because the two bonds experience distinct chemical environments; the upper bond is closer to the nitrogen lone pair. Consequently, this asymmetry accounts for the deviation of the NCO group from linearity, demonstrating that the two bonds in C=O in the HNCO minimum-energy structure are not strictly equivalent. In essence, although these bonds are conventionally described as equivalent, the two bent bonds in C=O experience subtly different chemical environments, leading to appreciable differences in their electronic interactions.

This analysis is possible only within the bent-bond framework, which allows differentiation between the above- and below-plane regions of the molecule.^80^ Consequently, the framework introduces a form of stereochemical character to unsaturated systems, stemming from the quasi-tetrahedral nature of the bound atoms.^81^

Furthermore, as demonstrated by the relaxed PES scan, when the HNC angle is constrained to 180°, the molecule adopts a fully linear structure (**HNCO** ^**lin**^), with the nitrogen lone pair converting into an additional N–C bond, as indicated by the IBOs. SCGVB calculations likewise predict a fully linear structure that is consistent with a zwitterionic form characterized by an N≡C triple bond with a positively charged nitrogen and an O^–^ center bearing three lone pairs.

The bottom right plot of Figure 2(C) illustrates how *E*[I] evolves during the transformation from **HNCO** ^**bent**^ to **HNCO** ^**lin**^. Notably, the conversion of the nitrogen lone pair into the NC(3) bent bond and the formation of the sigma CO(1) bond from the CO(2) bent bond contribute to the stabilization of **HNCO** ^**lin**^ relative to that of **HNCO** ^**bent**^. However, three factors collectively favor the stabilization of the bent structure: (a) the destabilization of the two remaining bent bonds in N≡C, namely, NC(1) and NC(2), in **HNCO** ^**lin**^; (b) the destabilization resulting from the conversion of CO(1) into the oxygen lone pair O­(lp3); (c) the destabilization of the other two oxygen lone pairs that occurs concomitantly with the formation of O­(lp3). These combined effects lead to a preference for the **HNCO** ^**bent**^ structure. The *E*[I] values for all orbital pairs are provided in Table S6.

As a final comment, the *trans*-bent minimum **HNCO** ^**bent**^, whose formation is attributed here to the distinct chemical environments of the bent bonds in C=O, can also be understood in terms of the asymmetric resonance hybrids proposed in Scheme 1. In this framework, the electron delocalization that enables the interconversion of these resonance structures closely resembles the anomeric effect, an interaction that, in other contexts, accounts for the quasi-planarity of amides when depicted with a bent bond representation.^80^

In summary, both MO and VB approaches yield consistent connectivity of the atoms: the bent minimum is characterized by two double bonds (N=C and C=O), while the linear structure features a triple N≡C bond alongside a single C–O bond, with the oxygen atom accommodating three lone pairs. These findings suggest that as nitrogen adopts a more *sp*-like hybridization and its bond with carbon strengthens, the N–C bond length decreases, and the overall molecular structure becomes more linear. Conversely, a weakening of the NC bond leads to a longer N–C distance and enhanced localization of the lone pair on nitrogen, ultimately resulting in a bent NCO configuration.

### Substituent Effects

Building on the consistency observed between the SCGVB and IBO analyses for HNCO, we extended our investigation to analogous RNCO systems to assess the impact of various substituents R on the isocyanate group. This investigation serves to test our hypothesis that the hybridization state controls the stereoelectronic preferences of the isocyanate moiety.

For practical purposes, we focus on IBO analysis. Figure 3(A) displays the IBOs for NCO^–^. These orbitals clearly illustrate an N≡C triple bond, with one oxygen lone pair aligned parallel to each of the π bonds (plus an additional lone pair not shown in the figure). These findings underscore the dominant role of the configuration depicted in Scheme 1(A) for NCO^–^.

**Figure 3:**
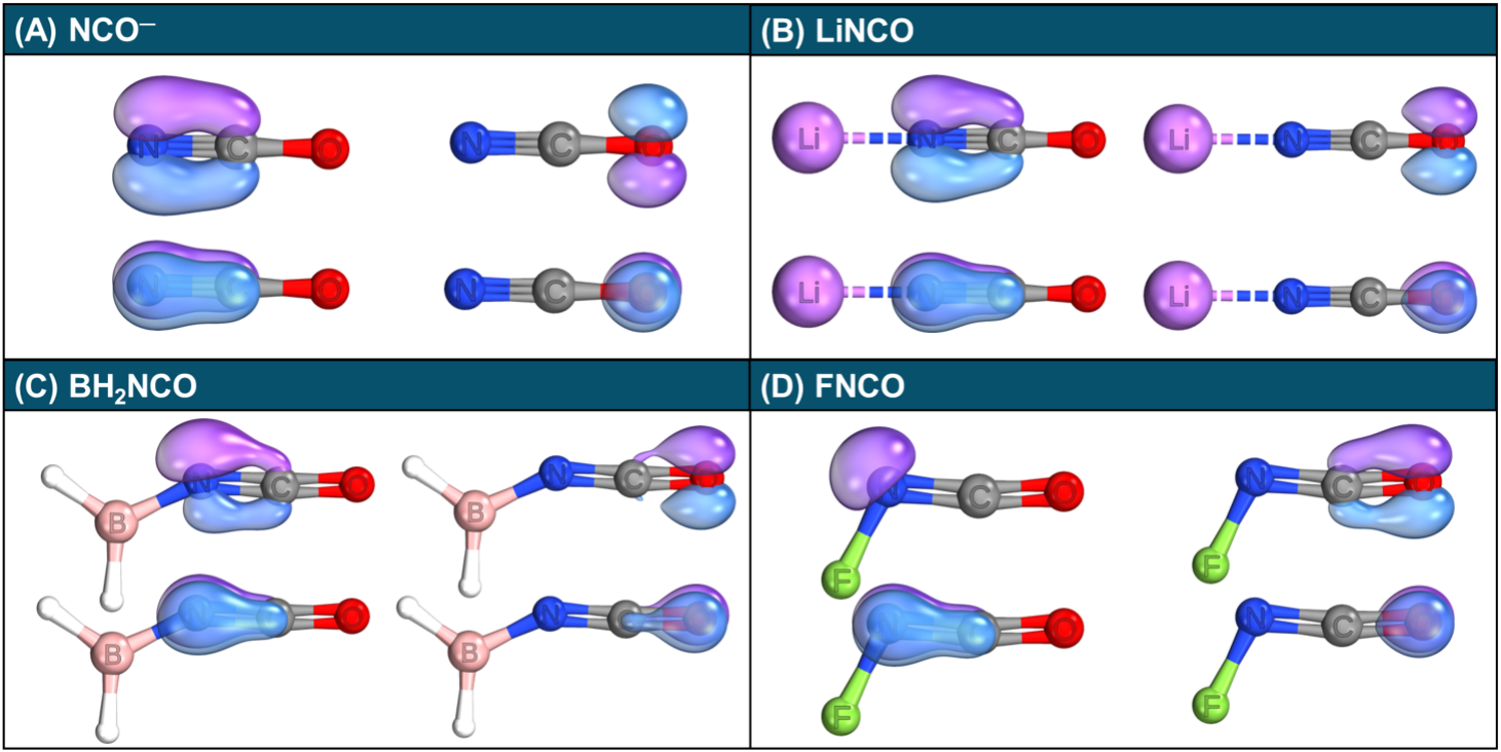
IBOs of the π and lone orbitals calculated at the ωB97M-D4/def2-TZVPP level for (A) NCO^–^, (B) LiNCO, (C) BH_2_NCO, and (D) FNCO. Orbital iso-surfaces visualized with threshold = 50%.

Figure 3(B–D) illustrates how different substituents influence the electronic structure of the isocyanate group in RNCO systems. The addition of Li^+^ to NCO^–^ yields a fully linear LiNCO structure that preserves the characteristic N≡C triple bond, supporting the notion that electron-donating groups, such as the Li atom, enforce the bonding scenario depicted in Scheme 1(A). In contrast, for FNCO (R = F), the linear RNCO arrangement is disrupted, as indicated by a strongly nonlinear FNC bond angle of 110.0°. Here, the loss of the NC triple bond and the emergence of a nitrogen lone pair, which interacts unfavorably with the CO framework, lead to a bent NCO structure. An intermediate case is observed for R = BH_2_, where the IBO analysis reveals an orbital with mixed nitrogen lone pair and C–N π-bond character, resulting in a wider R–N-C angle of 150.7°, compared to R = F, and a slightly bent NCO moiety.

Overall, these findings demonstrate that moving from electron-donating to electron-withdrawing substituents significantly impacts the nitrogen lone pair in the isocyanate group, as evidenced by both the orbital visualizations and variations in structural parameters (Table S7). These orbital interactions are further reflected in the structural parameters. For the representative cases of R = Li, BH_2_, and F, the N–C bond length increases from 1.193 to 1.202 and 1.246 Å, respectively.

The correlation matrix in Figure 4(A) encompasses geometric parameters, the NCO bending vibration (ν_bend_), and partial charges for the various R substituents (optimized geometries and vibrational frequencies are given in Tables S8 and S9, respectively). Closer inspection of this matrix reveals consistent trends among molecular descriptors such as bond lengths, angles, vibrational frequencies, and charge distributions, which illuminate the behavior of the isocyanate group under different substitutions. In Figure 4(B), several key correlations are highlighted. The leftmost plot (i) shows that as the RNC angle increases, the NCO angle approaches 180°, which is consistent with the conventional understanding that a shift in nitrogen hybridization from *sp* ^2^-like (approximately 120°) to *sp*-like (approximately 180°) results in N–C bond shortening. Additionally, the combined trends in plots (ii) and (iii) indicate that electron-withdrawing groups tend to induce more bent overall structures, as seen in the HNC angle, and result in a more pronounced bending of the NCO moiety, as evidenced by both the reduced NCO angle and the softening of the NCO bending vibrational mode.

**Figure 4:**
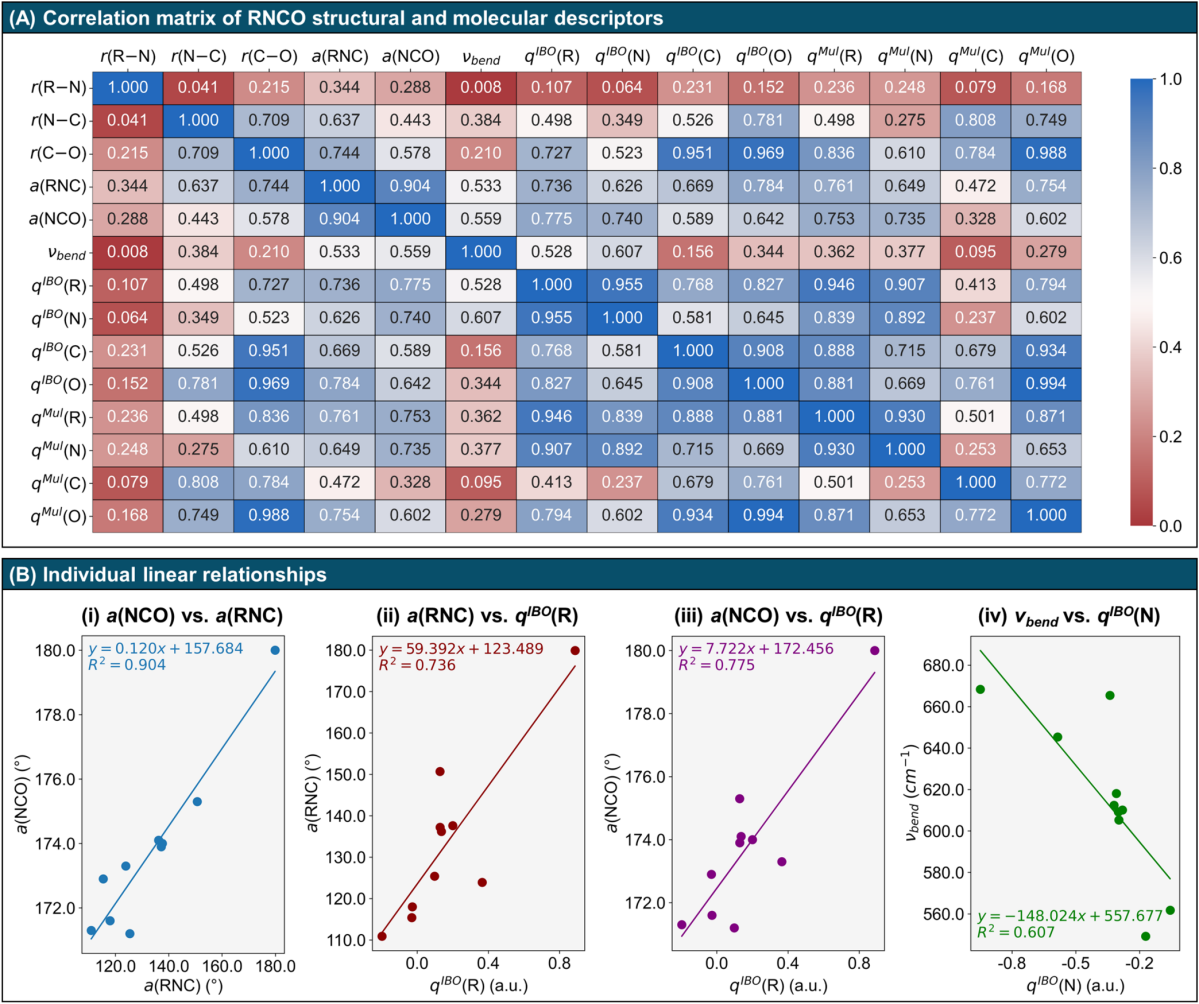
Correlation between molecular parameters of isocyanates (RNCO) for R = H, Li, F, BH_2_, CH_3_, NH_2_, HO, NO_2_, CH_2_CH, and Ph. (A) Correlation matrix showing the coefficient of determination of the linear regressions between bond lengths, bond angles, NCO bending modes, and partial charges computed using the IBO and Mulliken schemes. (B) Individual linear correlations between: (i) the RNC and NCO angles, (ii) the RNC angle and the IBO partial charge on the substituent R, (iii) the NCO angle and the IBO partial charge on the substituent R, and (iv) the NCO bending mode and the IBO charge on the nitrogen atom of the isocyanate group.

This depiction of the substituent effect, which influences the localization of the lone pair or its delocalization into a π-bond, aligns with the reactivity data reported by Wolf et al.^34^ and Nagy et al.^82^ They observed that electron-withdrawing groups, such as fluorine, tend to lower the energy barrier for proton transfer through the N=C bond. This behavior is consistent with the well-established basic chemistry principle that lone pairs are more likely to interact with protons than π-bonds.^83,84^

Finally, plot iv of Figure 4(B) illustrates how the stiffness of the NCO bending vibration is influenced by charge distribution, particularly over the nitrogen atom. Electron-donating groups tend to increase vibrational stiffness, whereas electron-withdrawing groups make the vibration more flexible. Both the localization of the nitrogen lone pair and the stiffness of the NCO group are directly related to the reactivity of isocyanates. These features should be carefully considered when attempting to modulate the reactivity of this functional group.

Table S10 summarizes the calculated partial charges obtained from IAO and Mulliken population analyses for all of the studied structures. In all cases, the carbon atom is partially positive. While the nitrogen and oxygen charges are comparable, electron-donating substituents make oxygen more negatively charged than nitrogen, whereas electron-withdrawing groups render nitrogen more negatively charged than oxygen. This trend underscores the influence of substituent electronic effects on the isocyanate charge distribution.

## Conclusions

In this work, we computationally investigate the stereoelectronic effects underlying the distorted *sp* carbon geometry in the isocyanates. Our study combines high-level CCSD­(T) calculations with both IBO and SCGVB analyses, revealing a consistent picture of the bonding in HNCO. In the bent minimum, the molecule is best described by two double bonds (N=C and C=O), whereas a fully linear structure corresponds to a resonance form featuring an N≡C triple bond alongside a single C–O bond with oxygen accommodating three lone pairs. Interference energy analysis further demonstrates that quantum interference plays a dominant role in stabilizing the bent structure by modulating orbital interactions and hybridization. Indeed, in the absence of interference, the NCO motif in HNCO would be completely flat. Furthermore, the analysis also reveals that the two banana bonds in the isocyanate C=O group are not equivalent. This inequivalence originates from the asymmetric electronic environment induced by the neighboring nitrogen lone pair, which weakens one of the bonds and promotes the overall bending of the NCO group.

Extending the investigation to RNCO systems reveals that the nature of the substituent markedly influences the isocyanate group. Electron-donating substituents, for example, the Li atom in LiNCO, enforce a linear configuration reminiscent of the NCO^–^ anion, whereas electron-withdrawing groups, such as the F atom in FNCO, disrupt this linearity by localizing the nitrogen lone pair and inducing a pronounced bending of the NCO moiety. These trends are corroborated by variations in key structural parameters—bond lengths, bond angles, and vibrational frequencies.

Overall, the findings presented herein provide a comprehensive mechanistic picture of the stereoelectronic origins of the isocyanate quasi-linearity. They underscore the critical role of orbital interactions and substituent effects in dictating the hybridization state and overall geometry of the isocyanate group, which, in turn, have significant implications for its reactivity. Future investigations may further explore how these stereoelectronic factors influence reaction pathways and the design of isocyanate-based materials.

## Supplementary Material



## Notes

Additional data, Cartesian coordinates of the optimized geometries, absolute energies, vibrational frequencies, and further details of the SCGVB calculations (PDF)
