# Supplementary material for: To Bend or Not to Bend: Revealing the Stereoelectronic Origin of the Distorted sp Carbon in Isocyanates
Source: J Phys Chem A. 2025 Aug 13;129(34):7751–60. doi: 10.1021/acs.jpca.5c02484 (PMC12400430; doi:10.1021/acs.jpca.5c02484)
Supplement: Supplementary file 1 [file jp5c02484_si_001.pdf]

Supporting Information

To Bend or Not to Bend: Revealing the Stereoelectronic Origin of the Distorted *sp* Carbon in Isocyanates

Lucas Araujo†, Felipe Fantuzzi*‡, Thiago M. Cardozo*¶ and Lars V. Schäfer*†*

† Center for Theoretical Chemistry, Ruhr University Bochum, Universitätsstr. 150, 44801 Bochum, Germany

‡ Chemistry and Forensic Science, School of Natural Sciences, University of Kent, Park Wood Rd, Canterbury CT2 7NH, UK

¶ Instituto de Química, Universidade Federal do Rio de Janeiro, Av. Athos da Silveira Ramos, 149, CT, A-622, Cid. Univ., Rio de Janeiro, RJ 21941-909, Brazil

Table of Contents

1. CCSD(T): Scan and Optimizations S2

2. CASSCF: Active Space S4

3. SCGVB: σ–π vs. Bent Bonds S5

4. SCGVB: GPF-EP S8

5 DFT: Geometric Parameters and Partial Charges S9

1. CCSD(T): Scan and Optimizations

Table S1: CCSD(T)/aug-cc-pVTZ relaxed PES scan varying the HNC angle (in degrees) and the IAO partial charges for each geometry.

angle HNC	Energy (a.u.)	q(H)	q(N)	q(C)	q(O)
115.00000000	-168.44536508	0.367234	-0.635281	0.697431	-0.429385
118.42105263	-168.44578949	0.370392	-0.635284	0.697923	-0.433031
121.84210526	-168.44594457	0.373650	-0.634819	0.698185	-0.437015
125.26315789	-168.44586313	0.376991	-0.633935	0.698261	-0.441317
128.68421053	-168.44557876	0.380371	-0.632681	0.698177	-0.445867
132.10526316	-168.44512533	0.383739	-0.631067	0.697967	-0.450640
135.52631579	-168.44453598	0.387075	-0.629301	0.697676	-0.455450
138.94736842	-168.44384269	0.390286	-0.627257	0.697248	-0.460277
142.36842105	-168.44307589	0.393356	-0.625044	0.696731	-0.465044
145.78947368	-168.44226415	0.396251	-0.622723	0.696144	-0.469672
149.21052632	-168.44143423	0.398914	-0.620382	0.695503	-0.474034
152.63157895	-168.44061132	0.401378	-0.618033	0.694842	-0.478187
156.05263158	-168.43981919	0.403592	-0.615772	0.694183	-0.482002
159.47368421	-168.43908006	0.405538	-0.613661	0.693549	-0.485426
162.89473684	-168.43841451	0.407203	-0.611763	0.692966	-0.488406
166.31578947	-168.43784114	0.408576	-0.610132	0.692456	-0.490900
169.73684211	-168.43737650	0.409649	-0.608808	0.692041	-0.492882
173.15789474	-168.43703376	0.410420	-0.607843	0.691730	-0.494307
176.57894737	-168.43682394	0.410884	-0.607254	0.691539	-0.495169
180.00000000	-168.43675326	0.411039	-0.607060	0.691475	-0.495455

Table S2: Cartesian coordinates of the optimized geometries and absolute energies with and without constraints at the CCSD(T)/aug-cc-pVTZ level.

**HNCO**			
**Energy**	-168.445947008642		
**O**	-1.17383430098042	-0.00838920826016	-0.00000000000014
**C**	-0.00396321399300	-0.04011775634208	0.00000000000027
**N**	1.21070068568635	0.09546880224379	-0.00000000000010
**H**	1.84017782928707	-0.69052183764154	-0.00000000000003
**$HNCO^{bl}$**			
**Energy**	-168.444297082987		
**O**	-1.03966559277827	-0.19791241083878	-0.00000000031306
**C**	0.09384227991061	0.09462980313926	0.00000000062718
**N**	1.27493494253324	0.39945296814398	-0.00000000031461
**H**	2.00290176204943	-0.29574407702847	0.00000000000048
**$HNCO^{lin}$**			
**Energy**	-168.436753255753		
**O**	-1.22860218655102	0.14717589987099	0.00000001122915
**C**	-0.06686724130683	-0.06370943556638	-0.00000001816003
**N**	1.09767011391277	-0.27515875043245	0.00000000339309
**H**	2.07088011624507	-0.45186815237215	0.00000000353780

2. CASSCF: Active Space

To establish a reliable multireference description of HNCO, we began by exploring different approaches for constructing the active space in our CASSCF calculations. Methods tested included the use of natural orbitals as well as localized orbitals obtained through the Pipek–Mezey and Foster–Boys procedures. While all options produced satisfactory results for the occupied orbitals, the virtual orbitals proved more difficult to localize consistently. As a result, we opted to use canonical Hartree–Fock orbitals and systematically tested different active space compositions.

During this process, we noted a significant mixing of σ, π, and δ character in the lowest-lying virtual orbitals. We believe this mixing may be related to the bent geometries observed in the SCGVB calculations described in the following section. The final active space chosen comprised eight electrons in eight orbitals, and Figure S1 presents the resulting natural active orbitals obtained from the CASSCF(8,8) calculations for all three considered HNCO structures.

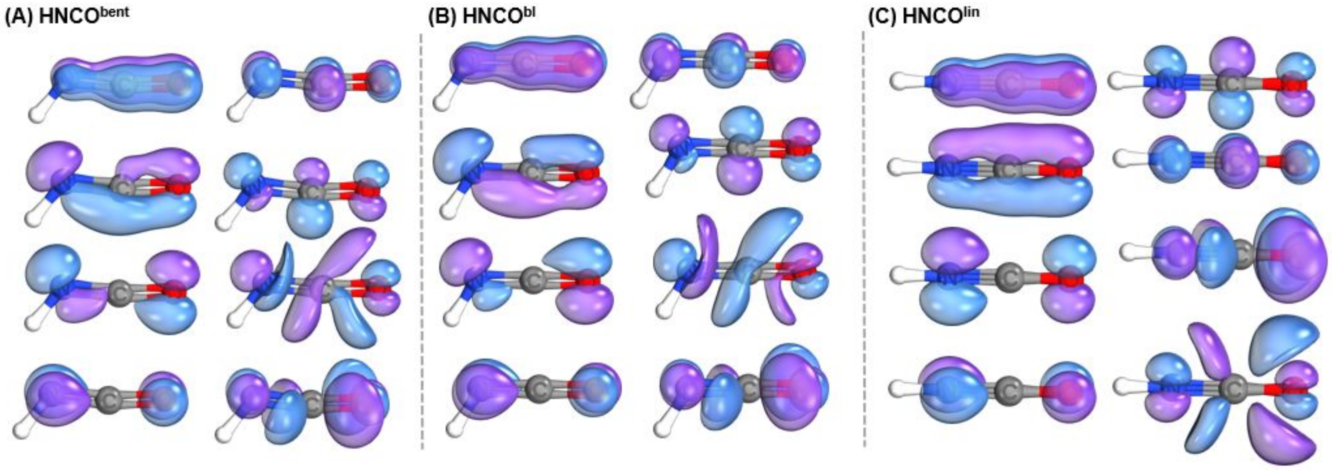

Figure S1: CASSCF(8,8)/cc-pVTZ natural active orbitals. (A) Bent minimum (**HNCO^{bent}**), (B) bent linear (**HNCO^{bl}**) and (C) constrained linear structure with optimized bonds (**HNCO^{lin}**).

3. SCGVB: σ–π vs. Bent Bonds

The HNCO molecule, used as a model for isocyanates, presented an unexpected challenge. In the Generalized Product Function (GPF), electrons are separated into strongly orthogonal (SO) groups by construction. When coupled into singlet electron pairs, this defines the so-called perfect pairing (PP) approximation with SO constraints. Here, we treated core electrons as a single Hartree–Fock group, while the valence electrons were assigned to SCGVB groups of two electrons each, forming what we refer to as SCGVB-PP.

The SCGVB-PP method exhibits a bias toward the σ–π separation, even though the bent-bond description yields slightly lower energies. Based on prior experience and available literature, the description of HNCO in terms of σ and π SCGVB-PP orbitals should not pose a problem. However, in most attempts during the VBSCF calculations, the orbitals naturally converged to the bent-bond representation, breaking the σ–π separation. To maintain this separation in the SCGVB calculations, Jacobi rotations between σ and π orbitals had to be disabled during wavefunction optimization, a procedure typically applied to more complex electronic structures such as benzene.

Figure S2 displays the orbital pairs within the σ–π separation for HNCO. Although the chemical structure, in terms of chemical bonds, aligns with the predictions from Intrinsic Bond Orbitals (IBOs) and the bent valence bond orbitals presented in the main text, the corresponding energetics are completely inadequate.

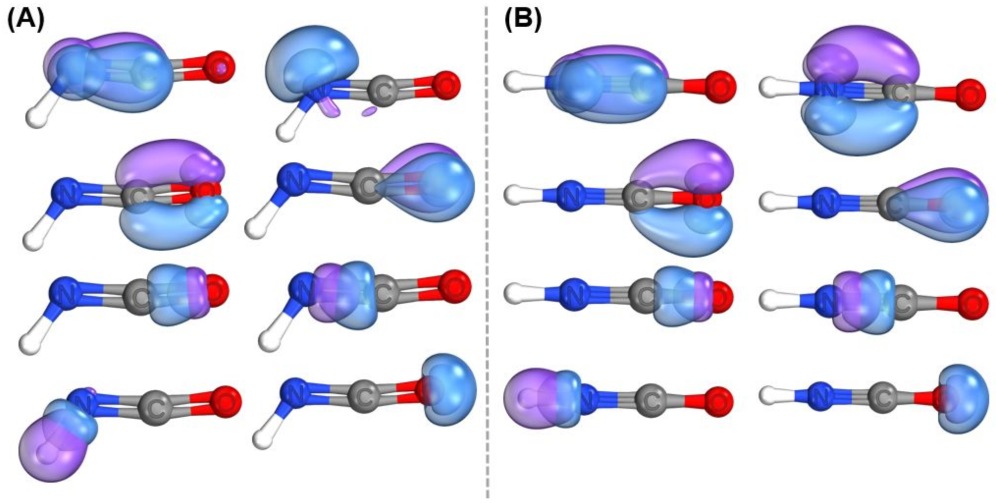

Figure S2: SCGVB-PP/cc-pVTZ//CCSD(T)/aug-cc-pVTZ orbitals pairs within the σ–π separation. (A) Bent minimum (**HNCO**$^{\mathbf{bent}}$) and (B) constrained linear structure with optimized bonds (**HNCO**$^{\mathbf{lin}}$).

As reported in Table S3, enforcing σ–π separation produces significant deviations in the predicted minimum and energy differences, in sharp contrast to the results from CASSCF(8,8) and CCSD(T). Upon closer inspection, these discrepancies can be attributed to a failure in the SCGVB-PP wavefunction when constrained to a σ–π framework in the bent minimum.

Table S3: Comparison between σ–π and bent-bond models for HNCO using SCGVB-PP/cc-pVTZ//CCSD(T)/aug-cc-pVTZ. Energy differences at the SCGVB(8).SCGVB-PP/cc-pVTZ, CASSCF(8,8)/cc-pVTZ and CCSD(T)/aug-cc-pVTZ level for the same geometries are also presented.

**HNCO**	**$HNCO^{bent}$**	**$HNCO^{lin}$**	**ΔE (kcal mol^{-1})**
**SCGVB-PP (σ–π)**	-167.89430865	-167.91846237	-15.16
**SCGVB-PP (bent)**	-167.94399606	-167.93214092	7.44
**SCGVB(8).SCGVB-PP(bent)**	-167.95356164	-167.93734533	10.18
**CASSCF(8,8)**	-167.94740105	-167.93615330	7.06
**CCSD(T)**	-168.44594702	-168.43675387	5.77

Although the SCGVB and CASSCF(8,8) results are in overall agreement, the comparison should be done carefully. In the CASSCF approach, the active space was selected based on π-character, whereas in SCGVB(8).SCGVB-PP we included all banana bonds in a single SC group, with the remaining valence electrons distributed in two-electron SCGVB-PP groups. Despite these differences, the orbital shapes obtained from SCGVB(8).SCGVB-PP calculations are qualitatively consistent with those from the simpler SCGVB-PP results.

Table S4 presents the Gallup–Norbeck spin-coupling coefficients, which are particularly appropriate in this context given the non-orthogonal spin bases used. Unlike the commonly employed Chirgwin–Coulson coefficients—which can produce unphysical results such as negative values or those greater than one—the Gallup–Norbeck coefficients are constrained to the [0,1] interval and thus provide a more meaningful representation of spin contributions.

Finally, we note that the SCGVB(8) wavefunction was initialised using the converged SCGVB-PP orbitals. Even so, the full spin-coupled optimisation proved difficult, with slower and less stable convergence. This further supports the practical advantage of using the SCGVB-PP approximation for this system.

Table S4: Gallup-Norbeck coefficients for the spin eigenfunctions used in the Spin-Coupled group of the SCGVB(8).SCGVB-PP/cc-pVTZ calculations.

**SC(8).SCGVB-PP/cc-pVTZ**	**Gallup-Norbeck Coefficients**		
**Coupling Pattern**	**$HNCO^{bent}$**	**$HNCO^{bl}$**	**$HNCO^{lin}$**
1-2 3-4 5-6 7-8	0.943	0.945	0.992
1-3 2-4 5-6 7-8	0.000	0.000	0.002
1-4 2-5 3-6 7-8	0.002	0.002	0.001
1-2 3-5 4-6 7-8	0.000	0.000	0.005
1-3 2-5 4-6 7-8	0.000	0.000	0.000
1-5 2-6 3-7 4-8	0.001	0.001	0.000
1-4 2-6 3-7 5-8	0.000	0.000	0.000
1-2 3-6 4-7 5-8	0.037	0.036	0.000
1-3 2-6 4-7 5-8	0.001	0.001	0.000
1-2 3-4 5-7 6-8	0.000	0.000	0.000
1-3 2-4 5-7 6-8	0.000	0.000	0.000
1-4 2-5 3-7 6-8	0.000	0.000	0.000
1-2 3-5 4-7 6-8	0.015	0.015	0.000
1-3 2-5 4-7 6-8	0.000	0.000	0.000

4. SCGVB: GPF-EP

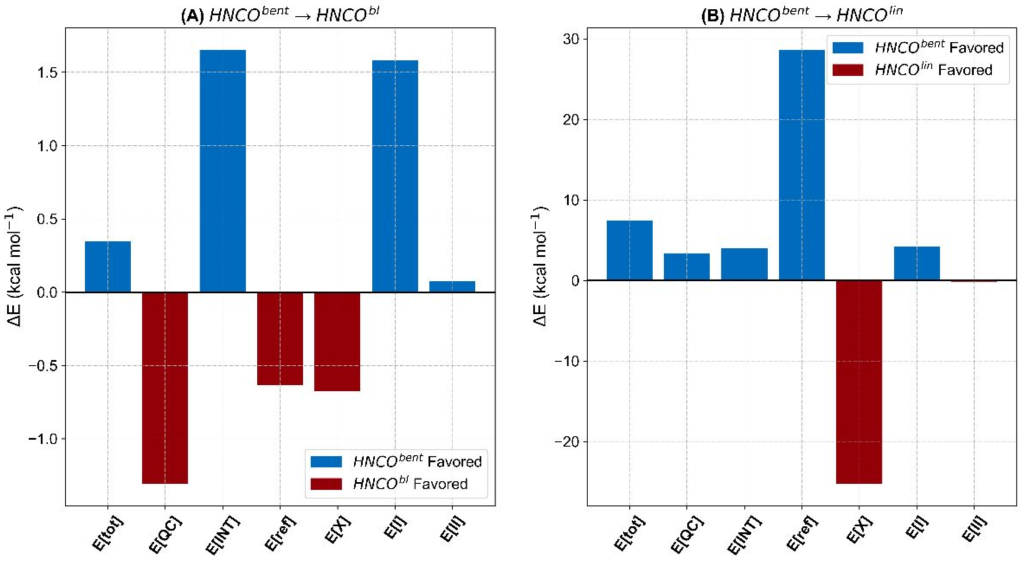

Figure S3: Interference energy analysis (kcal mol^{-1}) obtained at the SCGVB-PP/cc-pVTZ//CCSD(T)/aug-cc-pVTZ level for (A) **HNCO^{bent}** → **HNCO^{bl}** and (B) **HNCO^{bent}** → **HNCO^{lin}**.

Table S5: GPF-EP results for HNCO at the SCGVB-PP/cc-pVTZ//CCSD(T)/aug-cc-pVTZ level of theory.

	**HNCO^{bent}**	**HNCO^{bl}**	**HNCO^{lin}**
**E[TOTAL]**	-167.943996	-167.943446	-167.932141
**E[I]**	-1.263277	-1.260758	-1.256619
**E[REF]**	-164.930244	-164.931256	-164.884569
**E[X]**	-1.757562	-1.758635	-1.797818
**E[II]**	0.007086	0.007203	0.006865

Table S6: E[I] contribution for the various orbitals pairs of **HNCO^{bent}**, **HNCO^{bl}**, and **HNCO^{lin}** at the SCGVB-PP/cc-pVTZ//CCSD(T)/aug-cc-pVTZ level of theory.

**Orbital Pairs**	**HNCO^{bent}**	**HNCO^{bl}**	**Orbital Pairs**	**HNCO^{lin}**
HN(1)	-0.153810	-0.153381	HN(1)	-0.156354
N(lp)	-0.132299	-0.132415	NC(3)	-0.146861
NC(1)	-0.154942	-0.155441	NC(1)	-0.147184
NC(2)	-0.154979	-0.155233	NC(2)	-0.147108
CO(1)	-0.174840	-0.159083	O(lp3)	-0.154922
CO(2)	-0.170239	-0.182696	CO(1)	-0.194189
O(lp1)	-0.161082	-0.161252	O(lp1)	-0.155032
O(lp2)	-0.161086	-0.161257	O(lp2)	-0.154968

5. DFT: Geometric Parameters and Partial Charges

Table S7: Bond lengths (in Å), angles (in degrees) and bending modes for NCO (ν_{bend}, in cm^{-1}) for different groups (R) obtained from the ωB97M-D4/def2-TZVPP calculations.

R	r(R−N)	r(N−C)	r(C−O)	a(RNC)	a(NCO)	ν_{bend} (NCO)
**H**	1.004	1.211	1.161	123.9	173.3	645.31
**Li**	1.729	1.193	1.184	179.9	180	668.34
**F**	1.400	1.246	1.152	110.9	171.3	561.73
**BH_2**	1.414	1.202	1.161	150.7	175.3	665.44
**CH_3**	1.442	1.197	1.169	137.6	174.0	612.36
**NH_2**	1.429	1.213	1.163	125.4	171.2	610.10
**HO**	1.408	1.225	1.160	118.0	171.6	549.20
**NO_2**	1.432	1.24	1.145	115.4	172.9	618.07
**CH_2CH**	1.399	1.203	1.164	137.2	173.9	609.19
**Ph**	1.405	1.202	1.165	136.2	174.1	605.29

Table S8: Cartesian coordinates of the optimized geometries and absolute energies at the level ωB97M-D4/def2-TZVPP.

**HNCO**			
Energy	-168.800709737351		
O	-1.16472997753388	-0.01065067575447	0.00000011665350
C	-0.00368557826702	-0.03246463012093	-0.00000021830168
N	1.20122263460908	0.08692769672063	0.00000008120392
H	1.84027372349182	-0.68737282934523	0.00000002044427
**LiNCO**			
Energy	-175.780914995479		
O	-1.39005904463150	0.31001528730850	0.000395852
C	-0.24201661362376	0.01973988848628	0.000121085
N	0.91468488706258	-0.27273307383759	-0.00027763
Li	2.59047177119269	-0.70058210195719	-0.000239307
**FNCO**			
Energy	-267.991625106946		
O	-1.19517814151315	-0.06911550757456	-0.00000079815009
C	-0.04608150888781	0.01343353853356	0.00000149512906
N	1.16941623802593	0.28893294345498	-0.00000061207175
F	1.94492421467503	-0.87681141291397	-0.00000008490722

**BH_2NCO**			
Energy	-194.286158000809		
O	-1.36543043887310	0.51033614279128	0.17510074456915
C	-0.25922454038040	0.16634101172881	0.09747471329830
N	0.90919473962937	-0.11376959891603	0.07428066772011
B	1.96884411095569	-0.94721322068665	-0.35194969666444
H	1.73135830501924	-1.88196160220178	-1.04559514790144
H	3.07020601095619	-0.68485567459961	-0.00150869366069

**CH_3NCO**			
Energy	-208.127424883353		
O	-1.30544258684558	0.23844919227216	-0.17271588696831
C	-0.16768540636910	0.04549225326548	0.01496003761613
N	0.99003174583474	-0.06481044519446	0.29725972332024
C	2.12237411352065	-0.84584978267596	-0.13694165476907
H	1.92052288078428	-1.91096314342247	-0.04071955548300
H	2.37121867368926	-0.62409249803845	-1.17294030245389
H	2.97755178752875	-0.59871639417030	0.48354495464589

**NH_2NCO**			
Energy	-224.140841044996		
O	-1.18247237753042	0.64282028665568	0.08674883618706
C	-0.17917401921158	0.05509495022433	0.10306700434111
N	0.81519545582132	-0.61530393012981	0.28837899572717
N	1.89690281306479	-0.76539345379678	-0.63393941539502
H	1.97905088243502	-1.76421250443088	-0.78405635648447
H	2.72544543272785	-0.50412829040654	-0.11239647701486

**HONCO**			
Energy	-243.991073577117		
O	-1.18426247838613	0.08751523437823	0.11758983503168
C	-0.04079885999896	-0.02932321985277	-0.04197610415150
N	1.15594134762740	0.00173546887337	-0.30043087030666
O	1.95965977149528	-1.05520501093031	0.16661277254510
H	2.82618715560141	-0.79792512282452	-0.15798793873462

**NO_2NCO**			
Energy	-373.391937458991		
O	-1.16011336289370	-0.21158505914276	-0.20915821271857
C	-0.05056801535244	0.01736626813062	-0.04276077381680

N	1.11140777213189	0.41420272846209	0.12963181050635
N	2.09447094351926	-0.61497686448305	0.29216642700356
O	3.20634440101128	-0.21497065848534	0.45622630675304
O	1.71203826158369	-1.76292641448156	0.24852444227241
**CH_2CHNCO**			
Energy	-246.239640539849		
O	-1.38743983883462	0.10584307975851	0.01586681746461
C	-0.22452452464021	0.06071690655074	-0.01261930705934
N	0.97434309804835	0.13891359909242	-0.06758341475186
C	2.06588816530396	-0.72390326432671	0.07371845582132
H	3.01366805931743	-0.22561707015460	-0.06100228337718
**H**	1.04521578677554	-2.52301530543717	0.47942086419157
**H**	2.89577592855192	-2.59853737305298	0.43278220783896
**C**	1.99102112290861	-2.01847540313321	0.34348044769593
**PhNCO**			
Energy	-400.007165336116		
O	-4.31063807189781	-1.47797317323265	1.46821791568330
C	-3.39423636071713	-0.79359662189297	1.24706921322973
N	-2.51687708903353	0.00700412480182	1.06181804945340
C	0.68175949947837	-1.19446582267484	-0.34955943700779
C	-0.61570424242344	-1.21015527921016	0.13451935642353
C	-1.20437129541352	-0.02485344473461	0.56301940007827
C	-0.49588733948096	1.16750389501294	0.50576808966772
C	0.80154122651105	1.17222296012848	0.01943867698508
C	1.39521624834434	-0.00574455374124	-0.40948664186918
H	1.13592896596764	-2.11707518142374	-0.68131791918341
H	-1.17337482375032	-2.13497886784409	0.18201481123870
H	-0.97051393463501	2.07650159772502	0.84320885733514
H	1.34983824753669	2.10224435121333	-0.02352563679426
H	2.40684725653365	0.00150489065372	-0.78784669993424

Table S9: Vibrational frequencies (cm^{-1}) of the optimized geometries at the ωB97M-D4/def2-TZVPP level.

Molecule	Vibrational Frequencies (cm^{-1})
HNCO	568.53, 645.29, 806.35, 1351.92, 2338.89, 3703.56
LiNCO	114.18, 667.71, 668.34, 703.85, 1419.49, 2295.39
FNCO	213.78, 561.51, 731.41, 931.36, 1324.25, 2270.71
BH_2NCO	100.46, 156.20, 618.69, 665.44, 916.08, 968.45, 1038.96, 1267.39, 1585.78, 2375.06, 2617.20, 2708.83
CH_3NCO	32.24, 165.86, 612.36, 660.04, 896.98, 1130.46, 1153.94, 1457.41, 1485.30, 1505.25, 1524.30, 2372.37, 3065.73, 3129.69, 3156.96
NH_2NCO	42.03, 192.77, 610.06, 680.59, 901.60, 1080.46, 1336.27, 1460.29, 1676.71, 2311.64, 3505.45, 3574.22
HONCO	213.35, 231.14, 549.20, 717.08, 926.35, 1307.40, 1511.71, 2302.55, 3860.64
NO_2NCO	76.31, 154.51, 536.74, 563.09, 618.07, 780.18, 780.39, 909.48, 1330.26, 1372.75, 1711.63, 2310.12
CH_2CHNCO	102.07, 113.40, 497.42, 609.19, 663.23, 704.06, 873.38, 927.30, 1013.13, 1094.26, 1341.88, 1432.55, 1529.69, 1713.90, 2353.09, 3177.21, 3224.08, 3273.17
PhNCO	50.91, 88.49, 241.32, 383.89, 421.30, 474.83, 510.13, 605.29, 629.74, 669.09, 712.17, 781.39, 790.35, 859.63, 942.27, 1003.14, 1021.05, 1025.53, 1059.24, 1108.84, 1165.19, 1174.51, 1200.69, 1302.67, 1353.67, 1492.48, 1500.58, 1572.87, 1660.88, 1680.02, 2353.05, 3195.70, 3202.62, 3211.87, 3222.04, 3229.41

Table S10: Comparison of IAO and Mulliken Partial Charges (in atomic units) for different substituents (R) computed at the ωB97M-D4/def2-TZVPP level.

	IAO Partial Charges				Mulliken Partial Charges			
R	q(R)	q(N)	q(C)	q(O)	q(R)	q(N)	q(C)	q(O)
H	0.365	-0.571	0.568	-0.362	0.176	-0.287	0.331	-0.22
Li	0.887	-0.874	0.475	-0.489	0.764	-0.699	0.292	-0.357
F	-0.197	-0.13	0.595	-0.268	-0.128	-0.106	0.368	-0.134
BH_2	0.128	-0.366	0.588	-0.349	0.035	-0.161	0.333	-0.206
CH_3	0.2	-0.35	0.543	-0.393	0.196	-0.228	0.288	-0.257
NH_2	0.098	-0.317	0.573	-0.355	0.119	-0.231	0.333	-0.221
HO	-0.027	-0.226	0.573	-0.32	0.008	-0.146	0.329	-0.191
NO_2	-0.03	-0.341	0.626	-0.255	-0.057	-0.213	0.378	-0.108
CH_2CH	0.129	-0.333	0.567	-0.363	0.132	-0.228	0.322	-0.226
Ph	0.136	-0.331	0.561	-0.366	0.15	-0.218	0.298	-0.229
